# Neddylation is required for presynaptic clustering of mGlu7 and maturation of presynaptic terminals

**DOI:** 10.1038/s12276-021-00585-z

**Published:** 2021-03-25

**Authors:** Minji Kang, DoEun Lee, Jae-man Song, Sunha Park, Da-ha Park, Sanghyeon Lee, Young Ho Suh

**Affiliations:** 1 Department of Biomedical Sciences, Seoul National University College of Medicine, Seoul, 03080 Republic of Korea; 2 Neuroscience Research Institute, Seoul National University College of Medicine, Seoul, 03080 Republic of Korea; 3 Transplantation Research Institute, Seoul National University College of Medicine, Seoul, 03080 Republic of Korea

**Keywords:** Cellular neuroscience, Molecular neuroscience

## Abstract

Neddylation is a posttranslational modification in which NEDD8 is conjugated to a target substrate by cellular processes similar to those involved in ubiquitination. Recent studies have identified PSD-95 and cofilin as substrates for neddylation in the brain and have shown that neddylation modulates the maturation and stability of dendritic spines in developing neurons. However, the precise substrates and functional consequences of neddylation at presynaptic terminals remain elusive. Here, we provide evidence that the mGlu7 receptor is a target of neddylation in heterologous cells and rat primary cultured neurons. We found that mGlu7 neddylation is reduced by agonist treatment and is required for the clustering of mGlu7 in the presynaptic active zone. In addition, we observed that neddylation is not required for the endocytosis of mGlu7, but it facilitates the ubiquitination of mGlu7 and stabilizes mGlu7 protein expression. Finally, we demonstrate that neddylation is necessary for the maturation of excitatory presynaptic terminals, providing a key role for neddylation in synaptic function.

## Nervous system: Nerve cell development

New insights into the role of a regulatory protein in nerve development and activity in the brain will increase understanding of the maturation of nerve cells and the pathology of neurodevelopmental disorders. Young Ho Suh and colleagues at Seoul National University College of Medicine in South Korea studied cultured rat nerve cells to investigate a process called neddylation, mediated by the protein NEDD8. Neddylation occurs when NEDD8 is attached to other proteins in a manner that regulates the cell proliferation, but details of neddylation’s effects have been elusive in nerve cells. The authors have now identified a specific receptor protein in nerve cell membranes as a target for neddylation. This process seems to be required for the maturation and activity of nerve endings called pre-synapses, which release neurotransmitters to send signals to other nerve cells.

## Introduction

The metabotropic glutamate 7 (mGlu7) receptor is a class III G protein-coupled receptor that regulates synaptic transmission and plasticity in the mammalian brain. mGlu7 is primarily localized in the presynaptic active zone, where it suppresses the release of neurotransmitters by acting as an autoinhibitory receptor^1–3^. Several studies have demonstrated that stable neuronal surface expression of mGlu7 is important for proper receptor function and is regulated by posttranslational modifications (PTMs) and receptor-scaffold protein interactions^4–9^. For example, mGlu7 ubiquitination and neuronal surface stability are regulated by Nedd4, a ubiquitin (Ub) E3 ligase recruited by adaptor β-arrestins^5^. Furthermore, recent studies regarding mGlu7 pathogenic variants identified in patients with neurodevelopmental disorders found that stable neuronal surface expression of mGlu7 is essential for axon outgrowth and presynaptic terminal development^10,11^.

Similar to ubiquitination, neddylation is a cellular process in which neural precursor cell expressed, developmentally down-regulated 8 (NEDD8) is conjugated to a target substrate. Neddylation is accomplished by three sequential enzymatic reactions mediated by different enzymes, including the NEDD8 E1-activating enzyme (NAE) (NAE1/UBA3 heterodimer), E2-conjugating enzymes (UBC12 or UBE2F), and E3 ligases such as RBX1/2, SMURF1, c-CBL, MDM2, TRIM40, RNF111, or IAPs. Deconjugation of NEDD8 is achieved by COP9 signalosome complex subunit 5 (CSN5), DEN1 (also known as NEDP1 or SENP8), or USP21^12^. The best-characterized target substrate of neddylation is the family of cullin scaffold proteins. Neddylation enhances the activity of cullin-RING Ub ligases (CRLs), leading to the ubiquitination and subsequent degradation of their substrates, which are involved in multiple biological processes, including cell cycle progression^13^. Although NEDD8 was first identified in the brain, very few neddylation substrates, including PSD-95, cofilin/actin-depolymerizing factor (ADF), parkin, and PINK, have been identified in neurons^14–18^. A recent elegant study revealed that neddylation is essential for clustering of the dendrite scaffold protein PSD-95 and is necessary for dendritic spine stability and maturation^17^. In addition, inhibition of neddylation in developing neurons impairs cytoskeletal organization, neurite outgrowth, and dendrite development. These consequences are thought to result from impaired neddylation of cofilin and ADP^18^. Parkin and PINK1 have also been shown to be neddylated, and neddylation increases parkin E3 ligase activity and PINK1 stability^16^.

In the present study, we discovered mGlu7 as a target substrate for NEDD8 conjugation in both heterologous cells and rat primary cultured neurons. We also found that endogenous mGlu7 neddylation was reduced by agonist stimulation. In addition, neddylation modulated the ubiquitination and stability of mGlu7. Moreover, we demonstrated that neddylation was necessary for the localization of mGlu7 in the presynaptic active zone and the maturation of excitatory presynaptic terminals. Our findings will provide greater insight into the essential roles of neddylation in neuronal synapses.

## Materials and Methods

### Antibodies, reagents, and plasmid constructs

The antibodies used in this study were purchased from the following commercial sources: NEDD8 (RRID: AB_1640720, Abcam, Cambridge, UK); HA (RRID: AB_2565006, Covance, Princeton, NJ, USA); mGluR7a (RRID: AB_310459) and VGLUT1 (RRID: AB_2301751) (Millipore, Burlington, MA, USA); pERK (RRID: AB_2095853, Cell Signaling Technology, Danvers, MA, USA); ERK (RRID: AB_2140110, Santa Cruz, Dallas, TX, USA); α-tubulin (RRID: AB_477583, Sigma-Aldrich, St. Louis, MO, USA); VGAT (RRID: AB_887873) and bassoon (RRID: AB_2290619) (Synaptic Systems, Goettingen, Germany); anti-rabbit IgG-HRP (RRID: AB_2313567, Jackson ImmunoResearch, West Grove, PA, USA); and His (RRID: AB_2536982), anti-mouse IgG-HRP (RRID: AB_2536527), and Alexa Fluor-conjugated secondary antibodies (RRID: AB_2534088, RRID: AB_2313567, RRID: AB_2534119, and RRID:AB_2535805) (Thermo Fisher Scientific, Waltham, MA, USA). Mouse anti-c-myc (9E10) antibody was generated from a 9E10 clone (RRID:CVCL_G671, ATCC, Manassas, VA, USA) in our laboratory. MLN4924 (Cat# A-1139) and L-AP4 (Cat# 0103) were purchased from Active Biochem (Maplewood, NJ, USA) and Tocris Bioscience (Bristol, UK), respectively. HA-NEDD8 (# 18711) and HA-Ub (# 17608) plasmid constructs were obtained from Addgene (Watertown, MA, USA). UBC12 C111S-His and NEDD8ΔGG were generated from UBC12 wild-type (WT)-His (# 20080, Addgene) and NEDD8, respectively, by site-directed mutagenesis.

### Primary cortical or hippocampal neuron culture

Primary cortical or hippocampal neurons were obtained from pregnant Sprague-Dawley rats on gestational day 18 (OrientBio, Seongnam, South Korea). All rats were kept and sacrificed in accordance with the guidelines of the Seoul National University Institutional Animal Care and Use Committee (Protocol No. SNU-161222-2-6). The hippocampi or cortices of the day 18 embryos of either sex were isolated in dissecting solution [Hanks’ Balanced Salt Solution with 10 mM HEPES and penicillin-streptomycin (Cat# 14170-161, Cat# 15630-080, and Cat# 15070-063; Thermo Fisher Scientific)]. The tissues were incubated for 12 min in dissecting solution with 0.05% trypsin and 0.157 mg/mL deoxyribonuclease I (Cat# T1005 and Cat# D5025; Sigma-Aldrich) at 37 °C. The trypsinized tissues were dissociated using a fire-polished Pasteur pipette. The triturated cells were then plated on poly D-lysine-coated culture plates in serum-free neurobasal medium with B-27 supplement (Cat# 21103-049 and Cat# 17504-044; Thermo Fisher Scientific) and 1% L-glutamine (Cat# G7513; Sigma-Aldrich). Neurons were maintained by supplementation with fresh neurobasal medium every 2–3 days.

### Western blotting, immunoprecipitation, and surface biotinylation assay

Primary cultured neurons or transfected HEK 293 T cells were lysed in TN lysis buffer (50 mM Tris-HCl, pH 8.0, 150 mM NaCl, 1% Triton X-100, and 0.1% SDS) with protease inhibitor cocktail (Cat# 11873580001, Roche, Basel, Switzerland) and phosphatase inhibitor cocktail (Cat# P3200-005, GenDEPOT, Katy, TX, USA). The lysates were centrifuged at 20,000 × *g* for 15 min at 4 °C to remove insoluble materials. The resulting supernatants were mixed with 6× SDS sample buffer and denatured at 100 °C for 5 min or 42 °C for 20 min. For immunoprecipitation, the supernatant was incubated with 0.5 μg of anti-myc or anti-NEDD8 antibody for 1 h at 4 °C. The lysate-antibody mixture was added to 20 μL of Protein G Beads (Cat# 11243233001, Roche) and further incubated overnight at 4 °C with gentle rocking. The beads were washed four times with lysis buffer, mixed with 6× SDS sample buffer, incubated at 42 °C for 20 min, and then incubated at 80 °C for 3 min. The samples were resolved by SDS-PAGE and transferred to PVDF membranes (Cat# IPVH00010, Millipore). The membranes were then incubated in 5% nonfat skim milk dissolved in Tris-buffered saline with 0.1% Tween 20 (Cat# T1003, Anatrace, Maumee, OH, USA; TBST) for 1 h at room temperature (RT). The membranes were probed with primary antibodies overnight at 4 °C. After several washes with TBST, the membranes were incubated with HRP-conjugated secondary antibodies. The washed membranes were briefly immersed in a chemiluminescent substrate (Cat# 34580, Thermo Fisher Scientific) and exposed to X-ray film (Cat# CP-BU, AGFA, Mortsel, Belgium).

For the surface biotinylation assay, primary cortical neurons were incubated with 0.5 mg/mL membrane-impermeable EZ-Link Sulfo-NHS-SS-Biotin (Cat# 21331, Thermo Fisher Scientific) in PBS++ buffer (0.01 M phosphate-buffered saline, pH 7.4, 1 mM MgCl_2_, and 0.1 mM CaCl_2_) for 20 min at 4 °C with gentle rocking. The remaining biotin was quenched with 50 mM glycine in PBS++. The surface-biotinylated neurons were lysed, and the supernatant was incubated with 20 µL Streptavidin-Agarose Beads (Cat# 20347, Thermo Fisher Scientific) for 2 h at 4 °C. After washing the beads, the bound proteins were analyzed by western blotting.

### Receptor internalization assay and confocal microscopy

Primary hippocampal neurons plated on glass coverslips were transfected with myc-mGlu7 at 10-day in vitro (DIV). At DIV14, the neurons were incubated with 2 μg/mL anti-c-myc antibody for 10 min at RT to label surface-expressed mGlu7. The neurons were returned to the conditioned medium in the absence or presence of 400 μM L-AP4 at 37 °C for 15 min to allow receptor internalization. The neurons were fixed with 4% paraformaldehyde/4% sucrose in PBS for 20 min and blocked with 10% normal goat serum for 1 h. The surface receptors were labeled with Alexa Fluor 647 anti-mouse IgG (converted to red). The neurons were then permeabilized with 0.25% Triton X-100 for 5 min and blocked with 10% normal goat serum for 1 h. The internalized intracellular receptors were then labeled with Alexa Fluor 568 anti-mouse IgG (converted to green). The amount of internalization was quantified from the z-stacked maximum-projection images using MetaMorph software (RRID:SCR_002368, Molecular Devices, Sunnyvale, CA, USA) following the manufacturer’s instructions. The images were separated into red (surface-bound receptors after internalization) and green (internalized receptors) channels. A region of interest (ROI) per neuron was selected along the neurites, but the soma regions were excluded due to signal saturation. The upper and lower thresholds were set to remove saturated and background signals, respectively. The integrated intensities from each channel were calculated from the selected ROIs. The internalization rate was obtained by dividing the internalized signal intensity by the total signal intensity (the sum of the signal intensities of surface-bound receptors plus those of internalized receptors). The relative internalization rate was normalized to the WT internalization rate. For endogenous protein staining, the fixed and permeabilized neurons were incubated overnight with primary antibodies at 4 °C, washed with PBS three times, and incubated with Alexa Fluor secondary antibodies for 1 h at RT. Z-stacked maximum projection images were obtained using a Zeiss LSM 800 confocal microscope (RRID:SCR_015963, Carl Zeiss, Oberkochen, Germany).

To quantify synaptic maturation, Z-stack images were analyzed with Imaris software (RRID:SCR_007370, Bitplane, Zurich, Switzerland) using the FilamentTracer tool in autodepth mode. VGLUT1- or VGAT-positive puncta within 30 μm dendrites were masked using the Surface tool. The surface algorithm was used to calculate the number, area (size), and intensity of each punctum.

### Statistical analysis

The data within the bar graphs represent the mean and standard error of the mean (SEM) based on at least three independent experiments and are presented as a proportion of the control values. The significance of the differences between the mean values of the data sets was determined using Student’s paired (or unpaired, if indicated) *t* test or one-way ANOVA followed by Tukey’s post hoc test using GraphPad Prism (RRID:SCR_002798, San Diego, CA, USA) software. A *p*-value < 0.05 was considered statistically significant.

## Results

### mGlu7 is neddylated in heterologous cells and primary cortical neurons

**Fig. 1:**
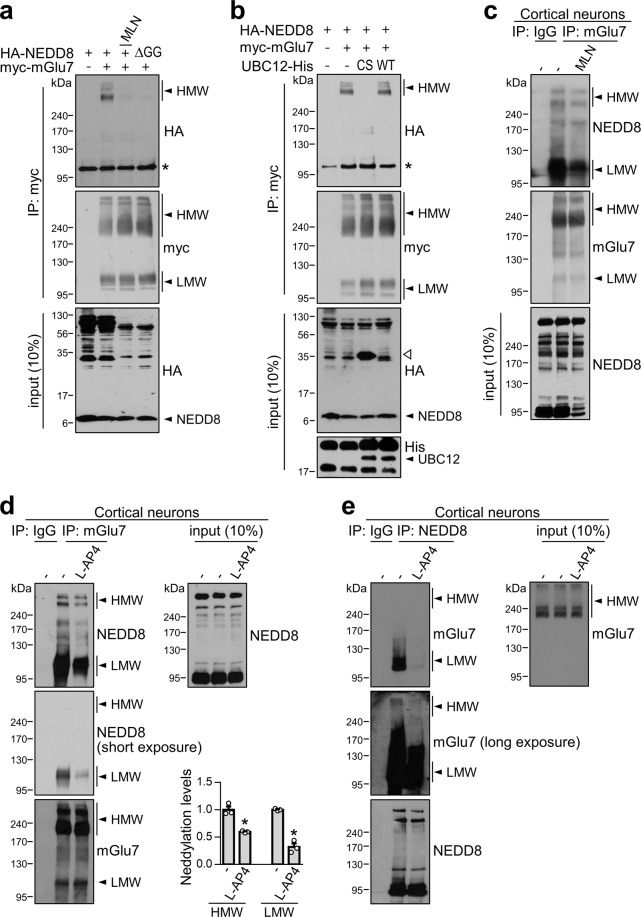
mGlu7 is neddylated in heterologous cells and primary cortical neurons. **a** HEK 293 T cells were cotransfected with myc-mGlu7 and HA-NEDD8 or HA-NEDD8ΔGG and treated with 1 μM MLN4924 or vehicle (DMSO) for 6 h. Thirty-six hours after transfection, cell lysates were immunoprecipitated with an anti-myc antibody. Western blotting was performed with anti-HA or anti-myc antibodies. *, nonspecific bands; MLN, MLN4924. **b** HEK 293 T cells were cotransfected with myc-mGlu7, HA-NEDD8, and UBC12 C111S-His or UBC12 WT-His. Immunoprecipitation and western blotting were performed as shown in panel **a**. An open triangle indicates the UBC12 C111S-NEDD8 complex. *, nonspecific bands. **c** Neddylation of endogenous mGlu7 is blocked by MLN4924. Primary cortical neurons (DIV14) were treated with 1 μM MLN4924 or vehicle for 6 h. Cell lysates were immunoprecipitated with anti-mGlu7 antibody and subjected to western blotting using the indicated antibodies. **d**, **e** Agonist stimulation reduces mGlu7 neddylation. Primary cortical neurons (DIV14) were treated with 400 μM L-AP4 for 5 min at 37 °C. Cell lysates were immunoprecipitated with an anti-mGlu7 antibody (**d**) or an anti-NEDD8 antibody (**e**). The immunoprecipitates were analyzed by western blotting. The bar graph with scatter plots shows the mean ± SEM (mGlu7 HMW, vehicle: 1.00 ± 0.06, L-AP4: 0.59 ± 0.01; mGlu7 LMW, vehicle: 1.00 ± 0.01, L-AP4: 0.32 ± 0.06; *n* = 3, *p < 0.05, Student’s *t* test).

To determine whether mGlu7 is a target protein of neddylation in heterologous cells, we cotransfected extracellular c-myc epitope-tagged mGlu7a (myc-mGlu7) and hemagglutinin epitope-tagged NEDD8 (HA-NEDD8) into HEK 293 T cells. The cell lysates were immunoprecipitated with anti-myc antibody and blotted with anti-HA antibody to detect the covalent attachment of HA-NEDD8 to mGlu7. The myc-mGlu7 bands existed mainly in the form of dimers (~240 kDa) and oligomers (larger than 240 kDa) [hereafter called mGlu7 high molecular weight (HMW)], and some existed in monomeric form (~110 kDa) [hereafter called mGlu7 low molecular weight (LMW)]^6^ (Fig. 1a). We observed robust neddylated mGlu7 HMW bands with apparent molecular weights above 240 kDa in the blot probed with the anti-HA antibody, whereas mGlu7 LMW was barely neddylated in HEK 293 T cells (Fig. 1a). To determine the specificity of the neddylated band, we utilized MLN4924, a selective inhibitor of NAE^19^, or HA-NEDD8ΔGG, a conjugation-defective mutant of NEDD8^20^. We found that treatment with 1 μM MLN4924 for 6 h or the expression of HA-NEDD8ΔGG markedly inhibited the neddylation of mGlu7 in HEK 293 T cells. As previously reported in the literature^17,21^, the total HA-NEDD8-conjugated protein levels were reduced by MLN4924 treatment or the expression of HA-NEDD8ΔGG in the input blot with anti-HA antibody, as the activation of NEDD8 was inhibited. Next, we examined the effect of the UBC12 C111S mutant, which is a dominant negative form of the NEDD8 E2-conjugating enzyme^19^, on the neddylation of mGlu7. Consistent with a previous report^22^, coexpression of UBC12 C111S markedly reduced the level of mGlu7 neddylation in HEK 293 T cells, which was similar to the effect of MLN4924, whereas coexpression of UBC12 WT did not have this effect (Fig. 1b). Since UBC12 C111S forms a stable complex with NEDD8, UBC12 C111S sequesters the NEDD8 monomers and prevents the subsequent transfer of NEDD8 to its targets^22^. This effect produced a prominent ~35-kDa band on the input blot that was detected by the anti-HA antibody (lane 3 in Fig. 1b).

Because overexpression of NEDD8 in heterologous cells may trigger neddylation by harnessing the ubiquitination machinery^12^, we investigated whether endogenous mGlu7 is neddylated without NEDD8 overexpression in rat primary cortical neurons. We immunoprecipitated endogenous mGlu7 with an anti-mGlu7 antibody using lysates of DIV14 cortical neurons and performed western blotting with an anti-NEDD8 antibody. We observed neddylated bands that were evident for both mGlu7 HMW and LMW, and these bands were reduced by MLN4924 treatment (Fig. 1c). In contrast to the results in heterologous cells, the neddylation of neuronal mGlu7 was much more prominent in the LMW form than in the HMW form (Fig. 1c). Of particular interest, the neddylated mGlu7 HMW and LMW band intensities were significantly reduced by treatment with L-AP4, a group III mGlu receptor agonist, to 59 ± 1% and 32 ± 6% that of the vehicle-treated control, respectively (Fig. 1d). Conversely, we detected neddylated mGlu7 HMW and LMW bands after immunoprecipitation with the anti-NEDD8 antibody in cortical neurons (Fig. 1e). Taken together, these results indicate that mGlu7 is a neddylation substrate under physiological conditions, and mGlu7 neddylation is dependent on its activation state.

### mGlu7 is neddylated on multiple lysine residues of the C-terminus and intracellular loops

**Fig. 2:**
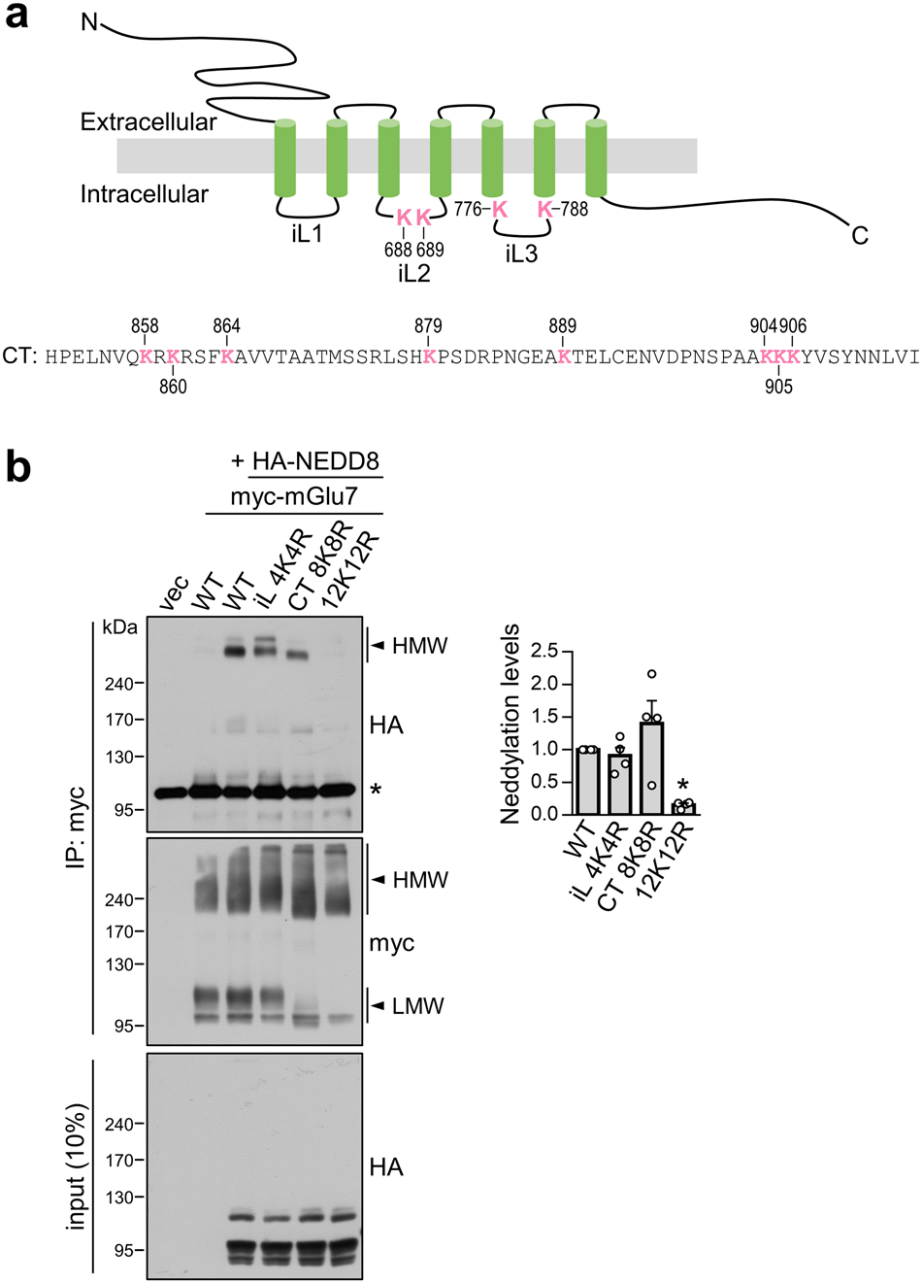
mGlu7 is neddylated at multiple lysine residues in both the CT and iL domains. **a** Schematic diagram showing the positions of the 12 lysine (K) residues in the rat mGlu7a amino acid sequence (GenBank Accession No. NP_112302.1). iL, intracellular loop; CT, C-terminal tail. **b** Analysis of mGlu7 neddylation residues. HEK 293 T cells were cotransfected with HA-NEDD8 and myc-mGlu7 WT, iL 4K4R, CT 8K8R, or 12K12R. The levels of mGlu7 neddylation were analyzed as shown in Fig. 1a. *, nonspecific bands. The bar graph with scatter plots shows the mean ± SEM normalized to WT (iL 4K4R, 0.91 ± 0.13; CT 8K8R, 1.40 ± 0.35; 12K12R, 0.16 ± 0.03; *n* = 4, *p < 0.05, n.s. indicates *p* > 0.05, one-way ANOVA followed by Tukey’s post hoc test).

Using site-directed mutagenesis methods, we previously analyzed the specific lysine residues to which Ub is covalently attached, but we were unable to identify the predominant ubiquitination sites in the mGlu7 C-terminus (CT) and intracellular loop (iL) domains^5^. To determine which lysine residues are responsible for NEDD8 conjugation of mGlu7, we utilized the mGlu7 iL 4K4R mutant, in which four lysine residues in mGlu7 iLs 2 and 3 are mutated to arginines, and the mGlu7 CT 8K8R mutant, in which eight lysine residues in mGlu7 CT are mutated to arginines^5^ (Fig. 2a). Similar to the mGlu7 ubiquitination data, neddylated mGlu7 was detected in both the mGlu7 iL 4K4R and mGlu7 CT 8K8R mutants, whereas the neddylated band was not detected in the mGlu7 12K12R mutant, a lysine null mutant of mGlu7 in which all twelve lysine residues are mutated to arginines (Fig. 2b). Thus, like ubiquitination, mGlu7 is likely to be neddylated at multiple lysine residues in both the CT and iLs.

### Neddylation is not involved in the constitutive and agonist-induced endocytosis of mGlu7

**Fig. 3:**
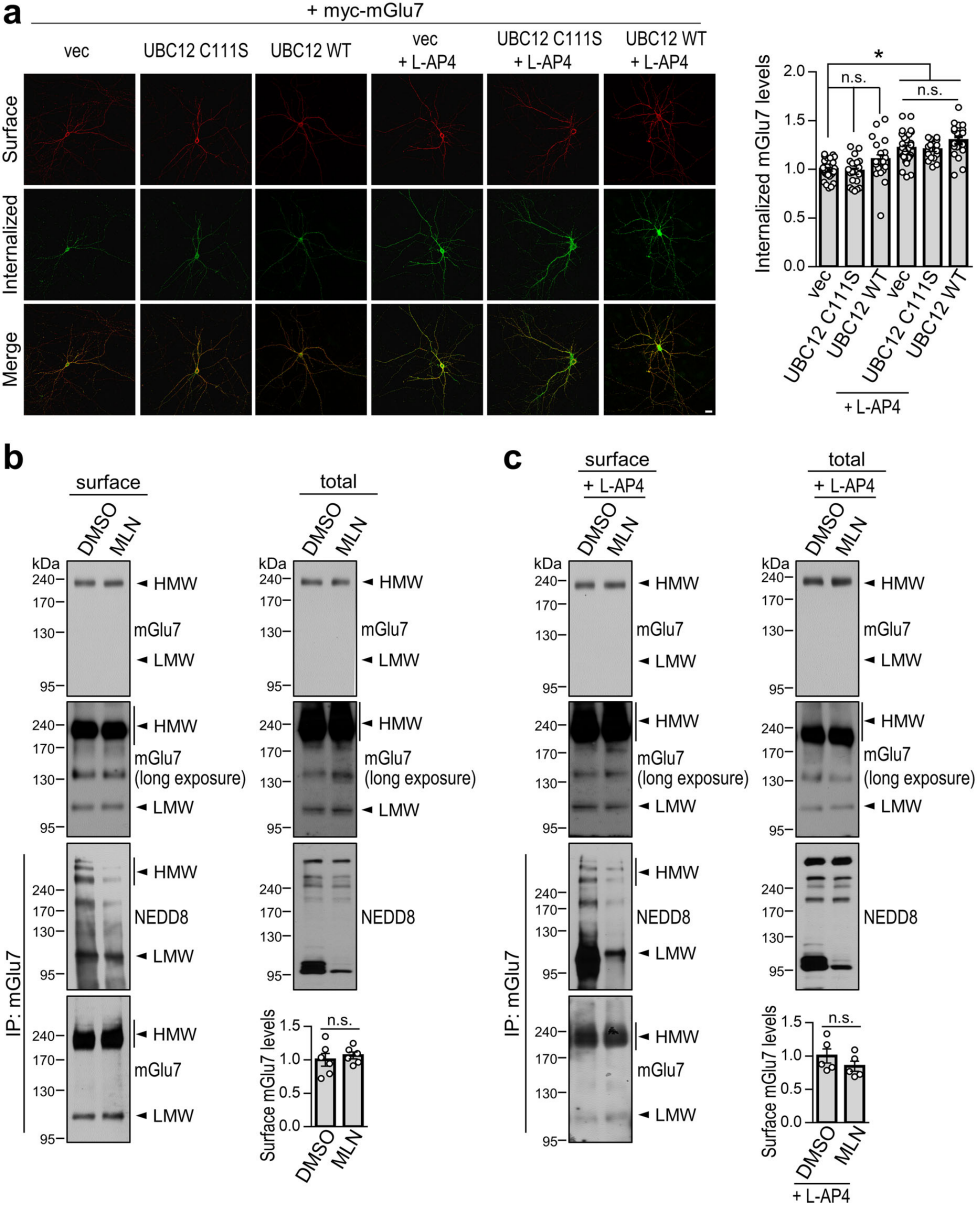
Effect of neddylation on mGlu7 endocytosis. **a** Primary hippocampal neurons (DIV10) were cotransfected with myc-mGlu7 and control vector (vec), UBC12 C111S, or UBC12 WT. Four days after transfection, the surface-expressed mGlu7 (red) and the internalized mGlu7 (green) were analyzed from Z-stacked maximal projection images using confocal microscopy. The representative images are presented. Scale bar, 20 μm. The summary quantification graph indicates the amount of internalized versus total (surface + internalized) mGlu7 using Metamorph software. The bar graph with scatter plots shows the mean ± SEM (vec, 1.00 ± 0.02; UBC12 C111S, 0.98 ± 0.03; UBC12 WT, 1.10 ± 0.05; vec + L-AP4, 1.22 ± 0.03; UBC12 C111S + L-AP4, 1.20 ± 0.02; UBC12 WT + L-AP4, 1.30 ± 0.04; *n* = 3, total neurons > 20; *p < 0.05, n.s. indicates *p* > 0.05, one-way ANOVA followed by Tukey’s post hoc test). **b**, **c** Surface biotinylation assay in cultured cortical neurons (DIV14). Neurons were pretreated with 1 μM MLN4924 or vehicle for 6 h and then unstimulated (**b**) or stimulated (**c**) with 400 μM L-AP4 for 15 min. Surface-expressed endogenous mGlu7 was biotinylated with membrane-impermeable Sulfo-NHS-SS-Biotin. After washing, the lysates were pulled down using streptavidin-agarose beads. The bead-bound proteins were detected by western blotting using an anti-mGlu7 antibody. The bar graph with scatter plots shows the mean ± SEM (**b**, DMSO, 1.00 ± 0.10; MLN, 1.07 ± 0.05; *n* = 6; n.s. indicates *p* > 0.05, Student’s *t* test. **c**, DMSO, 1.00 ± 0.11; MLN, 0.85 ± 0.08; *n* = 5; n.s. indicates *p* > 0.05, Student’s *t* test). To measure the effect of MLN4924, the bead-bound surface fraction was eluted using 50 mM DTT for 30 min at 50 °C. The eluted surface fraction was then immunoprecipitated using an anti-mGlu7 antibody and blotted with an anti-NEDD8 or anti-mGlu7 antibody.

It has been shown that neddylation of receptors such as epidermal growth factor receptor (EGFR) and transforming growth factor-β type II receptor (TGFβRII) can promote receptor endocytosis. After endocytosis, the receptors meet opposite fates in terms of receptor stabilization via neddylation. Neddylation facilitates the ubiquitination and degradation of EGFR while suppressing the ubiquitination and degradation of TGFβRII^23,24^. We first asked whether mGlu7 neddylation affects the endocytosis rate of mGlu7 by cotransfecting cultured hippocampal neurons with myc-mGlu7 and UBC12 C111S or WT. Surface-expressed myc-mGlu7 was labeled with an anti-myc antibody, and endocytosis was allowed at 37 °C for 15 min in the absence or presence of L-AP4. The mGlu7 remaining on the cell surface was labeled with Alexa Fluor 647-conjugated secondary antibody before permeabilization (converted to red), and internalized mGlu7 was visualized with Alexa Fluor 568-conjugated secondary antibody after permeabilization (converted to green). We observed that the agonist L-AP4 induced robust endocytosis of mGlu7, as demonstrated in previous studies^4,5,8,9^. Unlike the effect of L-AP4, the constitutive endocytosis of mGlu7 was not significantly altered by the expression of UBC12 C111S or WT (Fig. 3a). In addition, the agonist L-AP4-induced endocytosis of mGlu7 was not significantly altered by the expression of UBC12 C111S or WT (Fig. 3a).

Next, we performed a surface biotinylation assay to determine the neuronal surface expression of endogenous mGlu7 in cultured cortical neurons. Surface-expressed mGlu7 was labeled with membrane-impermeable Sulfo-NHS-SS-Biotin and pulled down using streptavidin-agarose beads. We observed no changes in the surface expression of mGlu7 after MLN4924 treatment for 6 h (Fig. 3b). In addition, MLN4924 treatment had little effect on L-AP4-induced mGlu7 endocytosis (Fig. 3c), suggesting that neddylation may not regulate the constitutive or agonist-induced endocytosis of mGlu7. We examined whether the neddylation of surface-expressed mGlu7 was reduced by MLN4924 treatment. After surface biotinylation of cortical neurons, the surface fraction was separated by pulling down using streptavidin beads and incubating the beads with 50 mM dithiothreitol (DTT) for 30 min at 50 °C. The isolated surface fraction was then immunoprecipitated using an anti-mGlu7 antibody and detected by blotting with an anti-NEDD8 antibody. We observed that MLN4924 treatment reduced the neddylation of surface-expressed mGlu7 under both constitutive and agonist-stimulated conditions (Fig. 3b, c).

### Neddylation facilitates mGlu7 ubiquitination and stabilizes mGlu7 protein expression but does not affect mGlu7-mediated ERK signaling

**Fig. 4:**
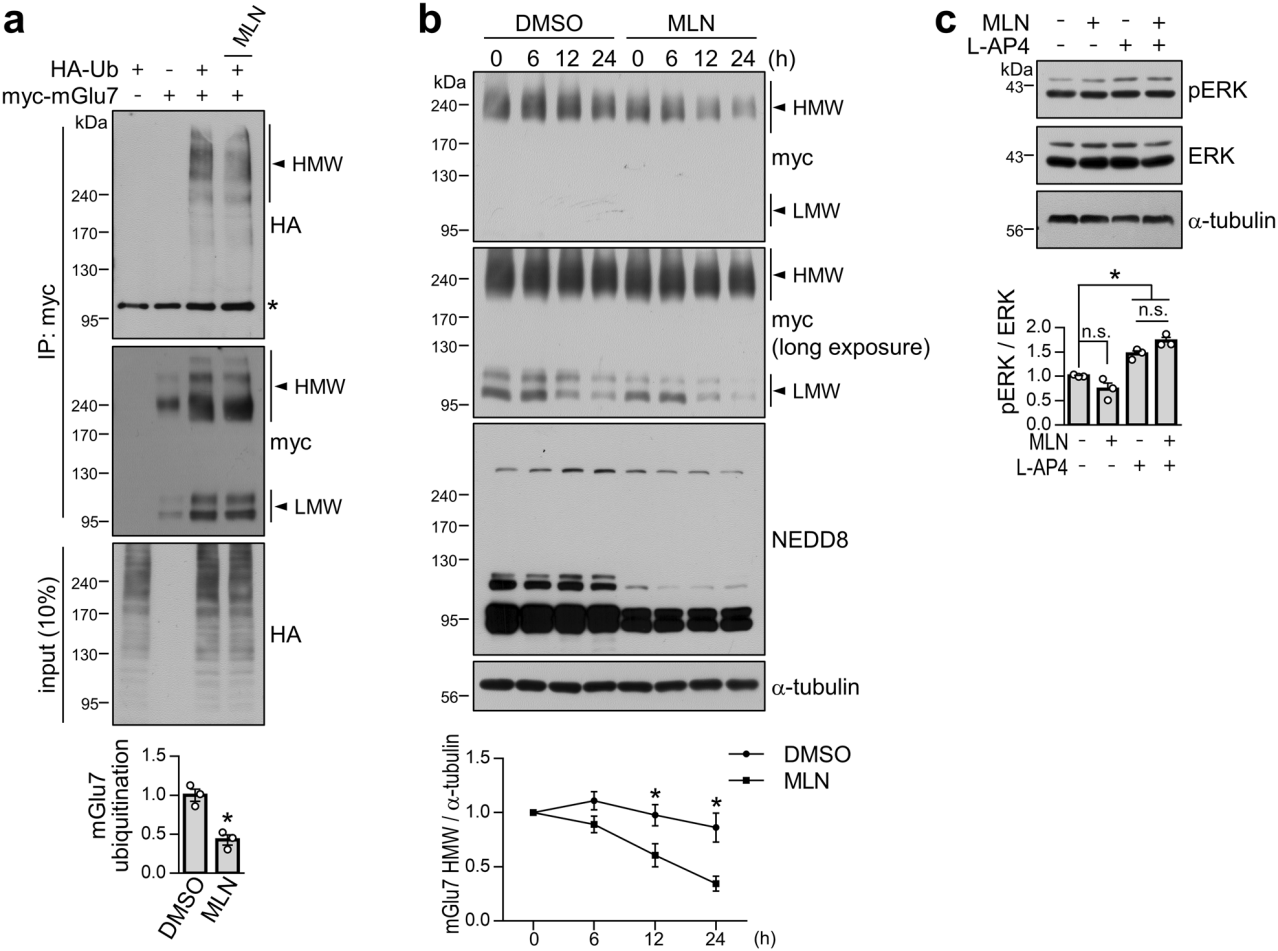
Effects of neddylation on mGlu7 ubiquitination, degradation, and signaling. **a** Neddylation facilitates mGlu7 ubiquitination. HEK 293 T cells were cotransfected with myc-mGlu7 and/or HA-Ub. Thirty-six hours after transfection, the cells were treated with 1 μM MLN4924 for 6 h and 1 mM L-glutamate for 5 min in the presence of MG132 and leupeptin. The cell lysates were immunoprecipitated with an anti-myc antibody. Western blotting was performed with the indicated antibodies. *, nonspecific bands; Ub, ubiquitin. The bar graph with scatter plots shows the mean ± SEM (DMSO, 1.00 ± 0.08; MLN, 0.43 ± 0.06; *n* = 3, *p < 0.05, Student’s *t* test). **b** Time-course experiment of the effect of neddylation on mGlu7 stability. HEK 293 T cells were transfected with myc-mGlu7 and HA-NEDD8 and pretreated with 1 μM MLN4924 for 12 h. The culture medium was changed to DMEM with 1% fetal bovine serum, and CHX was added for the indicated time periods; h, hours. Time-course expression of mGlu7 was quantified and is presented as the mean ± SEM normalized to 0 h (DMSO, 6 h: 1.10 ± 0.06, 12 h: 0.96 ± 0.07, 24 h: 0.83 ± 0.10; MLN, 6 h: 0.96 ± 0.09, 12 h: 0.66 ± 0.09, 24 h: 0.40 ± 0.08; *n* = 3, *p < 0.05, Student’s *t* test). **c** Neddylation is not required for mGlu7-mediated ERK signaling. Primary cortical neurons (DIV14) were treated with 1 μM MLN4924 and/or 400 μM L-AP4. Western blotting was performed with the indicated antibodies. The bar graph with scatter plots shows the mean ± SEM (vehicle, 1.00 ± 0.01; MLN, 0.73 ± 0.13; L-AP4, 1.45 ± 0.06; MLN + L-AP4, 1.72 ± 0.07; *n* = 3; *p < 0.05, n.s. indicates *p* > 0.05, one-way ANOVA followed by Tukey’s post hoc test).

We previously showed that upon agonist stimulation, mGlu7 is ubiquitinated and subsequently degraded via proteasomes and lysosomes^5^. To determine whether there is any crosstalk between neddylation and ubiquitination of mGlu7, we cotransfected myc-mGlu7 and HA-Ub into HEK 293 T cells and treated the cells with 1 mM L-glutamate for 5 min. The amount of mGlu7 ubiquitination was then evaluated by immunoprecipitation with an anti-myc antibody and western blotting with an anti-HA antibody. Consistent with a previous report^5^, we observed diffuse ubiquitinated mGlu7 signals. We found that MLN4924 treatment significantly decreased the band intensity of ubiquitinated mGlu7 to 43 ± 6% of that of DMSO treatment (Fig. 4a), indicating that neddylation facilitates agonist-induced mGlu7 ubiquitination.

Next, we investigated the effects of mGlu7 neddylation on protein degradation. HEK 293 T cells expressing myc-mGlu7 and HA-NEDD8 were pretreated with MLN4924 for 12 h, and cycloheximide (CHX) was then added during the indicated time course. We found that inhibition of neddylation by MLN4924 significantly enhanced mGlu7 degradation after 12 h of treatment with CHX (24 h treatment with MLN4924) (Fig. 4b), suggesting that neddylation stabilizes mGlu7 protein expression.

Since the mitogen-activated protein kinase (MAPK)/extracellular signal-regulated kinase (ERK) signaling pathway is thought to be downstream of mGlu7 in heterologous cells and neurons^5,25–27^, we examined the effect of MLN4924 on L-AP4-induced ERK signaling in primary cortical neurons. Consistent with previous literature, we observed that pERK levels were increased by L-AP4. However, we did not observe significant changes in pERK levels upon MLN4924 treatment (Fig. 4c), suggesting that neddylation is not related to mGlu7-mediated ERK signaling.

### Neddylation is necessary for the presynaptic clustering of mGlu7 to the active zone

**Fig. 5:**
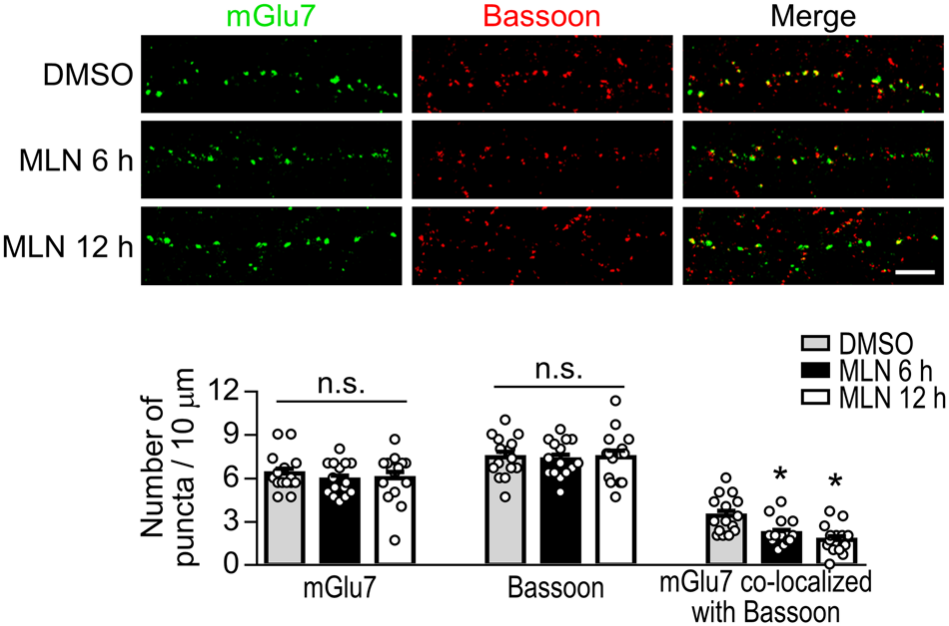
The distribution of endogenous mGlu7 is altered by MLN4924 treatment. Primary hippocampal neurons (DIV14) were treated with DMSO or 1 μM MLN4924 for 6 or 12 h. The neurons were fixed and coimmunostained with anti-mGlu7 and anti-bassoon antibodies. Representative images of neurites are presented. Scale bar, 5 μm. The summary quantification graph with scatter plots shows the mean ± SEM (mGlu7 puncta, DMSO: 6.33 ± 0.34, 6 h: 5.91 ± 0.29, 12 h: 6.02 ± 0.43; bassoon puncta, DMSO: 7.47 ± 0.37, 6 h: 7.31 ± 0.32, 12 h: 7.47 ± 0.45; mGlu7 puncta colocalized with bassoon, DMSO: 3.42 ± 0.33, 6 h: 2.20 ± 0.23, 12 h: 1.73 ± 0.25; *n* = 3, total dendrites = 15; *p < 0.05, n.s. indicates *p* > 0.05 versus DMSO, one-way ANOVA followed by Tukey’s post hoc test).

Neddylation has been shown to regulate PSD-95 clustering in the postsynaptic density of dendritic spines^17^. Thus, we investigated whether neddylation affects the localization of endogenous mGlu7 to the presynaptic active zone^3^. Primary hippocampal neurons were treated with MLN4924 for 6 or 12 h. We visualized endogenous mGlu7 and bassoon, a presynaptic active zone marker^28,29^, by immunostaining the neurons with anti-mGlu7 and anti-bassoon antibodies. We found that the number of endogenous mGlu7 puncta and the number of bassoon puncta did not change; however, the number of mGlu7 puncta that colocalized with bassoon was markedly reduced to 63 ± 7% or 52 ± 7% of the control value upon MLN4924 treatment for 6 or 12 h, respectively (Fig. 5). These results indicate that neddylation is required for the localization of mGlu7 in the presynaptic active zone.

### Neddylation is necessary for the maturation of excitatory presynaptic terminals

**Fig. 6:**
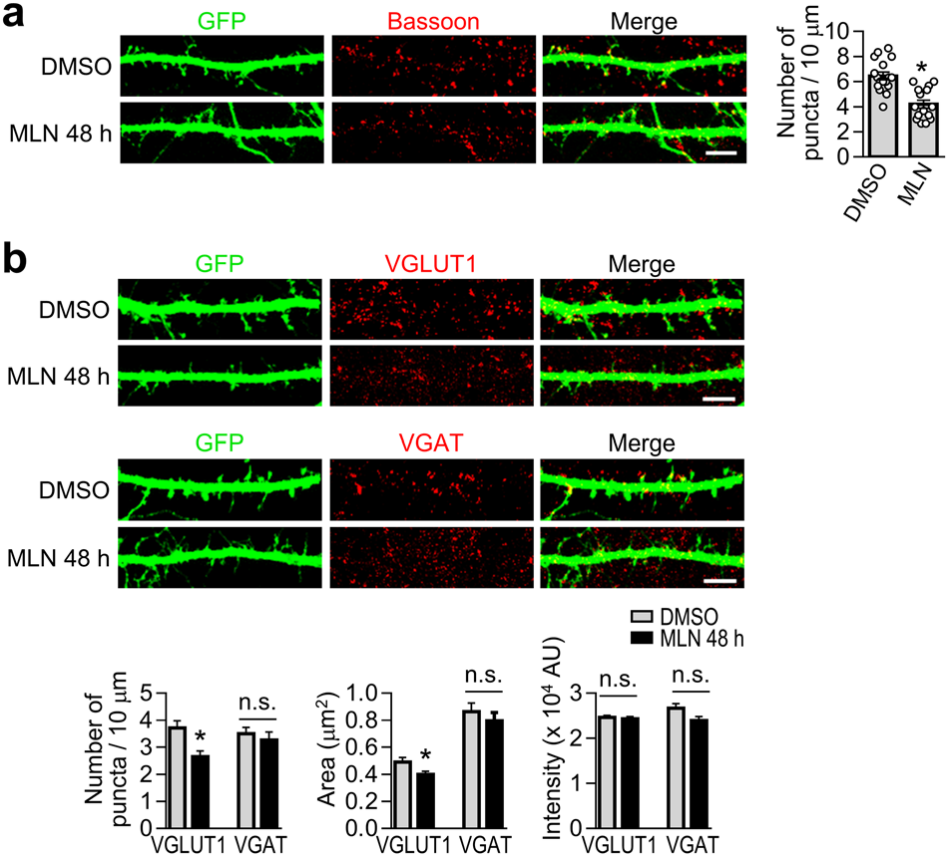
Neddylation is required for the maturation of excitatory presynaptic terminals. **a** Primary hippocampal neurons (DIV10) were transfected with EGFP. Forty-eight hours after transfection, the neurons were treated with DMSO or 1 μM MLN4924 for 48 h. At DIV14, neurons were immunostained with an anti-bassoon antibody. The number of puncta per 10-µm dendrite was quantified using the Imaris program. Scale bar, 5 μm. The bar graph with scatter plots shows the mean ± SEM (DMSO, 6.45 ± 0.31; MLN, 4.20 ± 0.32; *n* = 3, total dendrites = 15; *p < 0.05, Student’s unpaired *t* test). **b** As described in panel **a**, hippocampal neurons were immunostained with an anti-VGLUT1 antibody (upper panel) or anti-VGAT antibody (lower panel). The number of puncta per 10-µm dendrite (puncta density), size (area) of puncta, and intensity of puncta were quantified using the Imaris program. Scale bar, 5 μm. AU, arbitrary unit. Bar graphs show the mean ± SEM (VGLUT1 puncta, DMSO: 3.71 ± 0.26, MLN: 2.65 ± 0.21; VGAT puncta, DMSO: 3.50 ± 0.24, MLN: 3.27 ± 0.29; VGLUT1 area, DMSO: 0.49 ± 0.03, MLN: 0.40 ± 0.02; VGAT area, DMSO: 0.86 ± 0.06, MLN: 0.80 ± 0.06; VGLUT1 intensity, DMSO: 2.47 ± 0.04, MLN: 2.43 ± 0.05; VGAT intensity, DMSO: 2.67 ± 0.10, MLN: 2.39 ± 0.09; *n* = 3, total dendrites = 15, total number of puncta > 200; *p < 0.05, n.s. indicates *p* > 0.05, Student’s unpaired *t* test).

It has been demonstrated that neddylation is necessary for the maturation of postsynaptic dendritic spines^17^. However, whether neddylation affects the maturation of presynaptic terminals is still unclear. This prompted us to examine presynaptic terminal development via inhibition of neddylation. Forty-eight hours after treatment with MLN4924, primary hippocampal neurons were immunostained with an anti-bassoon antibody. We found that the number of bassoon-positive puncta along with GFP-positive dendrites was significantly reduced to 65 ± 5% that of the control upon long-term treatment with MLN4924 (Fig. 6a), indicating the necessity for neddylation in presynaptic terminal development.

Because bassoon is expressed in both excitatory and inhibitory presynaptic terminals^30^, we set out to determine which type of presynaptic terminal development is impaired by inhibiting neddylation. After primary hippocampal neurons were treated with MLN4924 for 48 h, the presynaptic terminals were visualized by immunostaining of the excitatory or inhibitory presynaptic terminal marker VGLUT1 or VGAT, respectively. We analyzed the number, size (area), and intensity of VGLUT1- or VGAT-positive puncta. Of particular interest, we found that MLN4924 significantly reduced the number and size (area) of VGLUT1-positive puncta to 71 ± 6% and 81 ± 5% that of the control treatment, respectively (Fig. 6b). The number and size of VGAT-positive synapses were not significantly altered, and the intensity of VGLUT1- or VGAT-positive puncta did not change (Fig. 6b). These results indicate that neddylation is necessary for the maturation of excitatory presynaptic terminals but not inhibitory presynaptic terminals.

## Discussion

Among several Ub-like proteins (UBLs), NEDD8 is the most abundantly expressed UBL in neurons and is widely expressed in the mouse brain^17^. However, the function and regulatory machinery of neddylation in the brain remain largely unknown. Recent studies have demonstrated that inhibition of neddylation reduces synaptic maturation, basal synaptic transmission, presynaptic release probability, postsynaptic AMPA and NMDA currents, surface expression of AMPA receptors, synaptic plasticity (both long-term potentiation and long-term depression), and cognitive functions^14,17,31,32^. While PSD-95 was previously identified as a critical substrate of neddylation in postsynaptic spines, in this study, we demonstrated that presynaptic mGlu7 is a target of neddylation, and inhibition of neddylation reduces the clustering of mGlu7 to the presynaptic active zone and reduces the maturation of excitatory presynaptic terminals.

Since NEDD8 is the closest homologous UBL to Ub, the activation and conjugation of NEDD8 can be achieved by utilizing the ubiquitination machinery. In particular, overexpression of exogenous NEDD8 can trigger the neddylation of substrates using the Ub E1-E2-E3 enzymes instead of NEDD8 E1-E2-E3 enzymes, leading to erroneous assignment of neddylation substrates^12^. Our previous study showed that mGlu7 is a ubiquitinated protein^5^, and we now show that mGlu7 is also a substrate of neddylation. However, our findings are unique to the neddylation process for the following reasons: i) Endogenous mGlu7 was neddylated without the overexpression of NEDD8 in neurons; ii) mGlu7 neddylation was blocked by MLN4924 treatment or the expression of UBC12 C111S, both of which are specific to the neddylation machinery; and iii) treatment of neurons with an mGlu7 agonist reduced mGlu7 neddylation while increasing mGlu7 ubiquitination^5^.

mGlu7 has a very low affinity for the intrinsic agonist L-glutamate, thereby acting only after high levels of the agonist are released into the synaptic cleft^2^. mGlu7 has been shown to inhibit neurotransmitter release upon the activation of G_i/o_ and the subsequent inhibition of calcium channels^33,34^. However, recent evidence has suggested that the prolonged activation of mGlu7 facilitates glutamate release via a G_i/o_-independent pathway through the translocation of Munc13 to the particulate fraction^35,36^. The deneddylated mGlu7 mislocalized from the active zone may no longer regulate synaptic vesicle release, thereby reducing the release probability. It is also plausible that the presynaptic machinery regulating synaptic vesicle release may be targets for neddylation.

It is well known that mGlu7 undergoes agonist-induced endocytosis^7^. PTMs such as phosphorylation at serine 862 and SUMOylation at lysine 889 on the mGluR7 C-terminus cause the retention of mGlu7 expression on the neuronal surface^4,9^. Unlike phosphorylation or SUMOylation, our findings suggest that neddylation does not regulate mGlu7 endocytosis. Instead, neddylation contributes to the stabilization of mGlu7 in the presynaptic active zone by prolonging the half-life of the receptor rather than by regulating receptor endocytosis. In addition, neddylation promotes the ubiquitination of mGlu7 while it stabilizes mGlu7 protein expression. This could be explained by the possibility that since deneddylated mGlu7 is displaced from the presynaptic active zone, deneddylation may reduce the availability of the Nedd4 E3 ligase-β-arrestin complex, which is involved in the ubiquitination of surface-expressed mGlu7 (both K63- and K48-mediated ubiquitination) in the active zone^5^.

We recently reported that the loss of mGlu7 on the cell surface via mGlu7 variant expression or mGlu7 knockdown results in the impairment of presynaptic terminal maturation^10^. Since neddylation is required for the localization of mGlu7 in the active zone, neddylated mGlu7 may contribute to presynaptic terminal maturation. However, it is unlikely that the neddylation of mGlu7 directly regulates presynaptic maturation. This is because mGlu7 is expressed at both excitatory and inhibitory presynaptic terminals^1,3^, but neddylated mGlu7 only affects the maturation of excitatory presynaptic terminals. In addition, the possibility that MLN4924 not only blocks mGlu7 neddylation but also blocks other unidentified target proteins necessary for synapse maturation at presynaptic terminals cannot be ruled out. Further elucidation of the various components involved in mGlu7 neddylation and the identification of other neddylation target substrates present at the synapse will be important for understanding the role of neddylation in synapse maturation and neurodevelopmental disorders.
